# A case of spinal myoclonus in a patient with elective cesarean section

**DOI:** 10.1186/s40981-018-0182-1

**Published:** 2018-06-18

**Authors:** Tohru Shiratori, Kunihisa Hotta, Masaaki Satoh, Naoko Kondo, Junji Ikeda, Shinji Sasao

**Affiliations:** 1 Department of Anesthesiology, Ina Central Hospital, 1313-1 Koshiroukubo, Ina, Nagano 396-8555 Japan; 2 Department of Anesthesiology and Critical Care Medicine, Jichi Medical University, 3311-1 Yakushiji, Shimotsuke, Tochigi 329-0498 Japan; 3 Department of Neurology, Ina Central Hospital, 1313-1 Koshiroukubo, Ina, Nagano 396-8555 Japan; 4 Department of Orthopedic Surgery, Ina Central Hospital, 1313-1 Koshiroukubo, Ina, Nagano 396-8555 Japan

**Keywords:** Movement disorder, Involuntary movement, Myoclonus, Local anesthetic, Spinal anesthesia, Neuraxial anesthesia, Cesarean section

## Abstract

**Background:**

Transient myoclonic involuntary movements, typically referred to as spinal myoclonus (SM), rarely develop in the extremities following neuraxial anesthesia (NA). NA indications in patients with history of SM following NA (SM-NA) are unknown.

**Case presentation:**

A 33-year-old woman developed SM-NA after elective cesarean section (CS). Approximately 130 min after spinal anesthesia induction, she began exhibiting involuntary movements, which became most severe after approximately 3 h. The involuntary movements gradually decreased without treatments and disappeared after approximately 5 h. The patient underwent CS on three occasions. The first CS (age, 29 years) was under a combination of spinal and epidural anesthesia. The third CS (age, 35 years) was completed using only spinal anesthesia. There were no neurological events during the postoperative courses for the first and third CS.

**Conclusions:**

SM-NA can unexpectedly occur, and history of SM-NA may not be contraindicative for repeated NA.

## Background

Myoclonus is defined as a sudden, brief, lightning-like involuntary muscle contraction originating from the central nervous system [1]. Transient myoclonic involuntary movements of the extremities, typically referred to as spinal myoclonus (SM), rarely develop following neuraxial anesthesia (NA) without consciousness disturbance [2]. SM is a non-generalized movement disorder that is presumed to originate from the spinal cord. Because SM develops following NA, it has been considered that NA may be responsible for the myoclonus. Furthermore, several articles related to SM following NA (SM-NA) speculate that local anesthetics interfere with the spinal cord, thereby leading to SM-NA [2–6]. However, the exact mechanism and prevalence of this movement disorder remain to be elucidated. Here, we report a case of SM-NA during the postoperative course of elective cesarean section (CS).

## Case presentation

A 33-year-old healthy woman (height, 159 cm; weight, 66 kg) underwent her second CS under spinal anesthesia. During the postoperative course, she experienced SM in the lower extremities. There were no preoperative neurological findings in her medical history, and preoperative laboratory examination results, including blood cell count, blood glucose level, and serum electrolyte concentrations, were within the normal range. Intrathecal administration of local anesthetic was performed at the L3/4 level using 0.5% hyperbaric bupivacaine (13 mg) without anesthetic premedication. Intermittent administration of ephedrine (at a total dose of 25 mg) was used to maintain the systolic blood pressure above 80 mmHg during the operation. Following the delivery of a healthy baby, droperidol (1.25 mg) was intravenously administered for nausea. Midazolam (2 mg) was also intravenously administered as a sedative at the patient’s request. Upon the completion of CS 67 min after induction of spinal anesthesia, the maximum level of sensory blockade was at the bilateral T2 level. Approximately 130 min after the intrathecal administration of local anesthetic, the patient complained of occasional myoclonic leg jerks in the maternity ward, which gradually increased in frequency and magnitude. The frequency and magnitude of the myoclonus were not constant, ranging from 2 to 30 times per minute. The patient’s pulse rate was 96 bpm, blood pressure was 120/78 mmHg, body temperature was 37.1 °C, and respiratory rate was 19 bpm, and her consciousness was clear. She presented severe postoperative lower abdominal pain associated with the myoclonic leg jerks; therefore, pentazocine (30 mg) and hydroxyzine (50 mg) were intravenously administered. No movement disorders were observed in the upper extremities. However, sensory perception in her lower extremities was not restored and she was unable to move her legs at will. The severity of the involuntary movements peaked at approximately 3 h after the induction of spinal anesthesia. The patient displayed bicycle-riding-like involuntary movements of the lower extremities; subsequently, these gradually decreased without treatments, and the sensation and voluntary movements in the lower extremities recovered. Approximately 5 h after the intrathecal administration of local anesthetic, the involuntary movements had almost disappeared. On postoperative day (POD) 1, neurological findings such as signs of meningitis, high fever, nuchal rigidity, impaired consciousness, or convulsions were absent. The patient was discharged on POD 8.

The patient underwent CS on three occasions. The first CS was at the age of 29 years and was performed under a combination of spinal and epidural anesthesia. Following placement of an epidural catheter at the T11/12 level, spinal anesthesia was performed at the L3/4 level using 0.5% hyperbaric bupivacaine (8.5 mg) and fentanyl (20 μg). Patient-controlled epidural analgesia was induced at a rate of 4 mL/h with 300 mL of 0.14% ropivacaine containing fentanyl (1.5 mg) and droperidol (7.5 mg). The third CS occurred at the age of 35 years and spinal anesthesia was performed at the L3/4 level using 0.5% hyperbaric bupivacaine (11.5 mg) and fentanyl (15 μg). Following delivery of a healthy baby, droperidol (1.25 mg) was intravenously administered for nausea. At the end of the CS, the maximum level of sensory blockade was at the bilateral T2 level. No postoperative neurological events occurred during the first or third CS.

## Discussion

The clinical course of the present case suggests some important clinical findings related to SM-NA: first, it is difficult to predict the occurrence of SM-NA; second, it is necessary to differentiate SM-NA from neuroleptic-induced extrapyramidal syndromes (EPS); and third, the bicycle-riding-like feature of myoclonic involuntary movement is a crucial symptom in the pathogenesis of the movement disorder.

As per our knowledge, there are no studies investigating the relationship between the history of NA and the occurrence of SM-NA. The clinical course described in the present case may be helpful in considering the prediction of SM-NA and indication of NA in patients with a history of SM-NA. The patient underwent spinal anesthesia on three separate occasions, with SM occurring only at the second occasion. According to previous case reports that recorded the patients’ past NA histories, some patients with SM-NA had previously undergone uneventful spinal anesthesia [2–4]. On the other hand, a case of repeated SM-NA was reported [5]. Inductive reasoning of the present and previous cases provides three clinical arguments useful for anesthetic practice: first, a history of uneventful spinal anesthesia does not guarantee a normal SM-NA-free recovery; second, patients with a history of SM-NA may be free from spinal myoclonus at the next NA; and third, it is unknown whether a history of SM-NA is a risk factor for repeated SM-NA. In our opinion, it is difficult to predict the occurrence of SM-NA and a history of SM-NA may not be contraindicative for repeated NA.

For the SM-NA diagnosis in the present case, it was important to differentiate SM-NA from drug-induced involuntary movements. Droperidol is a butyrophenone neuroleptic drug with antiemetic effects, which possibly induces EPS even at a low dose [7]. EPS caused by droperidol are typically classified into three types of involuntary movements: acute dystonia, parkinsonism, and akathisia. Acute dystonia includes muscle spasms in the tongue, face, neck, and back; parkinsonism includes bradykinesia, rigidity, and tremor; and akathisia includes motor restlessness [7]. The signs of SM-NA are lightning-like muscle jerks often confined to the extremities [2–6], whereas EPS is characterized by sustained muscle contractions causing twisting-like abnormal movements and postures, which may affect the facial muscles and may be generalized, involving the trunk as well as limbs [8, 9]. Although it may be difficult to differentiate droperidol-induced EPS under NA from SM-NA, careful observation of the involuntary movement patterns may make it possible to make the distinction.

SM is classified into segmental and propriospinal types. Segmental SM typically originates within a few or several adjacent spinal segments of the spinal cord, whereas propriospinal myoclonus refers to myoclonic involuntary movements that involve muscles innervated by many different segments [1, 10]. In our patient, SM was initially mild and subsequently evolved to severe movements. In previous cases, mild SM initially developed and evolved to jerky movements in the bilateral lower extremities [4, 5]. In severe cases, the SM increased at a frequency of more than 20 times per minute [2, 6] and evolved largely as dancing-like movements [3] or bicycle-riding-like movements, similar to that seen in the present patient. SM that resembles bicycle-riding-like involuntary movements seems to be a crucial symptom referred to as a severe form of SM-NA. In the present case, myoclonic involuntary movements occurred only in the lower extremities, suggesting the presence of segmental SM.

The present patient underwent spinal anesthesia on three separate occasions from which SM-NA occurred only at the second occasion. To our knowledge, no studies report the sporadic occurrence of SM-NA as described in the present case. SM-NA is a complication presumed to originate from the lumbar spinal cord involved with local anesthetic effects, which should be differentiated from drug-induced EPS. In this patient, midazolam was administrated during surgery. Some reports have suggested that the antiepileptic drug benzodiazepines may be useful in treating patients with SM-NA [10, 11]; however, a preceding administration of midazolam may contribute to few prophylactic effects for SM-NA. SM-NA sometimes presents in a severe form, which can cause unexpected perioperative complications. The clinical course of the present patient may be helpful in considering NA indications in patients with a history of SM.

### Conclusions

We encountered a patient who developed SM following spinal anesthesia during the postoperative course of CS. The movements were considered SM-NA rather than droperidol-induced EPS. The myoclonus gradually decreased without treatments. SM-NA can occur unexpectedly in patients without underlying diseases, and its occurrence is difficult to predict from NA history. Therefore, it is important to remember that SM is a potential complication following NA.

## Abbreviations

CS: Cesarean section
EPS: Extrapyramidal syndromes
NA: Neuraxial anesthesia
POD: Postoperative day
SM: Spinal myoclonus
SM-NA: Spinal myoclonus following neuraxial anesthesia
